# Improving the detection capability and efficiency of SARS-CoV-2 RNA specimens by the specimen turn-around process with multi-department cooperation

**DOI:** 10.3389/fpubh.2023.1294341

**Published:** 2024-01-05

**Authors:** Chenggui Liu, Wei Shen, Huiqiong Xie, Ying Li, Rong Cui, Rongcheng Wu, Li Xiao, Jing Li, Yanjun Guo, Yi Liao, Chonghui Zhao, Yunfei Xu, Qin Wang

**Affiliations:** ^1^Department of Clinical Laboratory, Chengdu Women's and Children's Central Hospital, School of Medicine, University of Electronic Science and Technology of China, Chengdu, China; ^2^Departments of Nursing, Chengdu Women's and Children's Central Hospital, School of Medicine, University of Electronic Science and Technology of China, Chengdu, China; ^3^Department of Specimen Sampling, Chengdu Women's and Children's Central Hospital, School of Medicine, University of Electronic Science and Technology of China, Chengdu, China; ^4^Department of Specimen Transportation, Chengdu Women's and Children's Central Hospital, School of Medicine, University of Electronic Science and Technology of China, Chengdu, China; ^5^Department of Information Technology, Chengdu Women's and Children's Central Hospital, School of Medicine, University of Electronic Science and Technology of China, Chengdu, China; ^6^Department of Medical Administration, Chengdu Women's and Children's Central Hospital, School of Medicine, University of Electronic Science and Technology of China, Chengdu, China; ^7^Department of Hospital Infection Control, Chengdu Women's and Children's Central Hospital, School of Medicine, University of Electronic Science and Technology of China, Chengdu, China; ^8^Departments of Medical Equipment, Chengdu Women's and Children's Central Hospital, School of Medicine, University of Electronic Science and Technology of China, Chengdu, China; ^9^Department of Clinical Laboratory, Sichuan Province Orthopedic Hospital, Chengdu, China

**Keywords:** COVID-19, SARS-CoV-2 RNA, detection capability, detection efficiency, process re-engineering

## Abstract

**Objective:**

Improving the detection capability and efficiency of severe acute respiratory syndrome coronavirus 2 (SARS-CoV-2) RNA specimens is very important for the prevention and control of the outbreak of Coronavirus disease 2019 (COVID-19). In this study, we evaluated the detection capability and efficiency of two outbreaks of COVID-19 before and after the process re-engineering in April and July 2022.

**Methods:**

This retrospective cross-sectional study involved 359,845 SARS-CoV-2 RNA specimens 2 weeks before and 2 weeks after the two outbreaks of COVID-19 in April and July. The number, transportation time and detection time of specimens, and the number of reports of more than 24 h were analyzed by SPSS software.

**Results:**

While 16.84% of people chose nasopharyngeal swabs (NPS) specimens, 83.16% chose oropharyngeal swabs (OPS) specimens to detect SARS-CoV-2 RNA. There were significant upward trends in the percentage of 10 sample pooling (P-10) from April before process re-engineering to July after process re-engineering (*p* < 0.001). Compared with April, the number of specimens in July increased significantly not only 2 weeks before but also 2 weeks after the outbreak of COVID-19, with an increase of 35.46 and 93.94%, respectively. After the process re-engineering, the number of reports more than 24 h in the 2 weeks before and after the outbreak of COVID-19 in July was significantly lower than that in April before process re-engineering (0% vs. 0.06% and 0 vs. 0.89%, both *p* < 0.001).

**Conclusion:**

The present study shows that strengthening the cooperation of multi-departments in process re-engineering, especially using the P-10 strategy and whole process informatization can improve the detection capability and efficiency of SARS-CoV-2 RNA specimens.

## Introduction

Coronavirus disease 2019 (COVID-19) is an ongoing global pandemic and highly infectious disease, mainly characterized by shortness of breath, fever, and pneumonia, which is caused by severe acute respiratory syndrome coronavirus 2 (SARS-CoV-2) (1, 2). SARS-CoV-2 is highly similar to severe acute respiratory syndrome coronavirus (SARS-CoV) and Middle East respiratory syndrome coronavirus (MERS-CoV) in genes and protein production levels, but there are still significant differences between them (3). SARS-CoV-2 RNA detection by real-time reverse transcriptase-polymerase chain reaction (RT-PCR) with specimens of nasopharyngeal swabs (NPS) or oropharyngeal swabs (OPS) or other respiratory swabs is one of the commendable measures for curbing the outbreak of COVID-19 (4–6). Early diagnosis of SARS-CoV-2 infection is the most critical step to prevent virus transmission (7, 8). In the fight against COVID-19, China formulated a series of effective management measures, including large-scale screening to ensure that all potentially infected persons are tested, isolated, hospitalized, or treated to control the outbreak of COVID-19 (9, 10).

Through nearly 3 years of prevention and control of COVID-19, our laboratory, like the laboratories in other Level III Grade A hospitals in China, is equipped with sufficiently trained personnel in RNA isolation and PCR analysis who have obtained the PCR work license. The RT-PCR reagents kit and consumable materials used to detect the SARS-CoV-2 RNA provided by commercial suppliers can also basically meet the needs of large-scale detection. However, after the outbreak of COVID-19 in Chengdu in April 2022, due to the difference between the number of specimens for large-scale detection of SARS-CoV-2 RNA and the number of specimens for routine detection of SARS-CoV-2 RNA, some problems were still exposed, which led to the long specimens’ turn-around time (TAT) and some TAT exceeded 24 h.

Several factors may affect the detection capability and efficiency of SARS-CoV-2 RNA in large-scale detection. First, the number of specimens exceeds the test load, which leads to some specimens being unable to complete the test within the specified time. Second, lower matching of information exchange between HIS and LIS and complex specimen turn-around process leads to difficulties in specimen handover and congestion. Third, the problem barcode that cannot be scanned and the problem specimen without a barcode cause work confusion, patient complaints, specimen resampling, etc., which leads to PCR testers spending a lot of time explaining and handling these problems. In addition, the number of hardware including PCR amplification instruments, nucleic acid extractors, etc. for detecting SARS-CoV-2 RNA is not enough, which leads to lower detection capability and efficiency of SARS-CoV-2 RNA.

In order to improve the capability and efficiency of SARS-CoV-2 RNA detection, a new SARS-CoV-2 RNA specimen turn-around process was established to meet both large-scale specimen tests and routine specimen tests by re-engineering with multi-department cooperation. In July 2022, when COVID-19 broke out again in Chengdu, we added some hardware and adopted this new process to classify and detect different medical types of people: A single specimen rapid test was used for patients with fever clinic and emergency, a single specimen routine test was used for people who must perform the test, and Pooling of 10 (P-10) samples strategy was used for people who volunteered to participate in the test. Moreover, informatization was used in the whole process of specimen sampling, specimen transporting, specimen searching, specimen testing, result uploading, etc. This new process has met the requirements of a large number of people with different medical types for detecting SARS-CoV-2 RNA specimens and has greatly solved the problems of different specimen numbers and shortage of medical personnel in the detection of SARS-CoV-2 RNA specimens.

## Methods

### Sources of data

In April and July 2022, there were two outbreaks of COVID-19 in Chengdu city, and both broke out for the first time on a Friday. The data were retrospectively collected at the Chengdu Women’s and Children’s Central Hospital, School of Medicine, University of Electronic Science and Technology of China, from 18 to 31 March 2022 (Friday to Thursday) to 1–14 April 2022 (Friday to Thursday), 2 weeks before and 2 weeks after the outbreak of COVID-19, and from 1 to 14 July 2022 (Friday to Thursday) to 15–28 July 2022 (Friday to Thursday), 2 weeks before and 2 weeks after the outbreak of COVID-19.

Subject information was obtained from medical staff (doctors, medical assistants, and laboratory technicians including PCR testers, nurses, and transport workers), which includes gender, age, medical type (in-patient, fever clinic, general out-patient, emergency patient, physical examination, and self-service through HIS and Tianfu health platform), name of SARS-CoV-2 RNA test item (single specimen rapid test, single specimen routine test, and mixed specimen test), and specimen type (NPS and OPS). The HIS automatically records the time of the doctor’s order, specimen sampling, specimen transportation, specimen reception, specimen testing, and report dispatch. The specimen TAT was calculated from specimen sampling to report dispatch, the transportation time of the specimen was calculated from specimen sampling to specimen reception, and the detection time of the specimen was calculated from specimen reception to report dispatch.

### Study population

The retrospective cross-sectional study was conducted from March 18 to April 14, 2022, and from July 1 to July 28, 2022, at Chengdu Women’s and Children’s Central Hospital, School of Medicine, University of Electronic Science and Technology of China. A total of 359,845 specimens of NPS or OPS from subjects used to detect SARS-CoV-2 RNA were enrolled in this study, including 54,416 specimens 2 weeks before the outbreak of COVID-19 and 78,831 specimens 2 weeks after the outbreak of COVID-19 in April, as well as 73,714 specimens 2 weeks before the outbreak of COVID-19 and 152,884 specimens 2 weeks after the outbreak of COVID-19 in July.

Specimens of subjects with some missing information including age, gender, specimen type, and the time of specimen turn-around process at each step, such as the time of doctor’s order and specimen sampling, were excluded from this study. Besides, environmental specimens for detecting SARS-CoV-2 RNA were also excluded from this study.

### The specimen turn-around process before and after re-engineering in April and July

Before the specimen turn-around process re-engineering, the application forms of SARS-CoV-2 RNA test items with the traditional method were ordered by doctors through the hospital information system (HIS), including single specimen rapid test or single specimen routine test. Specimens of NPS and OPS were sampled by nurses, transported by transport workers, and tested by PCR testers. The results of SARS-CoV-2 RNA were automatically uploaded to the Tianfu Health platform in Sichuan, China.

**Figure 1:**
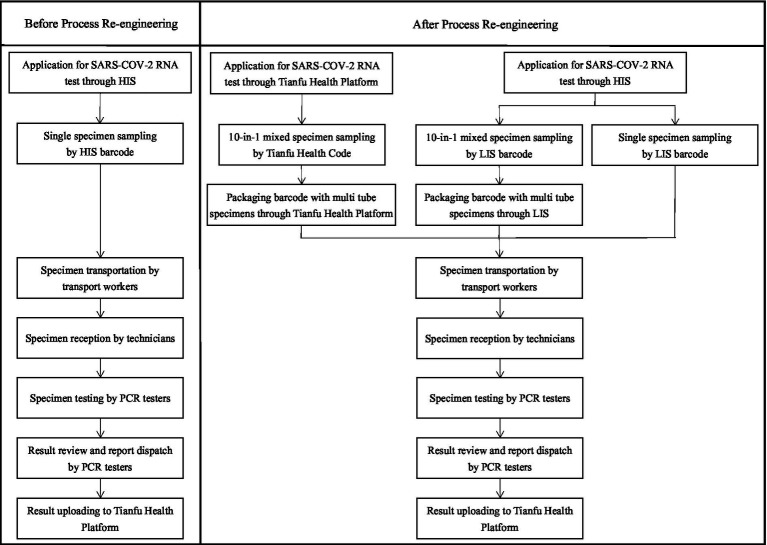
The specimen turn-around process before and after re-engineering in April and July.

On the basis of the traditional specimen turn-around process, several steps were re-engineered. First, the P-10 strategy (10 samples of NPS/OPS were pooled before the RNA extraction) was used as an additional option to detect the SARS-CoV-2 RNA of people who volunteered to participate in the test, which was ordered by the doctor from SARS-CoV-2 RNA items list in the doctors’ workstation of the HIS. The P-10 strategy was used for mass screening of populations and allowed for rapid and efficient epidemiologic screening while reducing testing costs. The limitation of this strategy was that it may have reduced sensitivity. Second, a large number of P-10 specimens of NPS or OPS containing volunteer information were rapidly sampled by scanning the rapid response code of the Tianfu Health platform (Tianfu Health Code) in Sichuan, China. Third, with the support of the information system, a box of specimens containing multiple specimen tubes with a packaging barcode was quickly transported and received by scanning this packaging barcode. Fourth, the frequency of specimen transportation was increased: specimens of fever and emergency should be delivered at least every 30 min, and routine specimens at least every 2 h. At the same time, the number of hardware for detecting SARS-COV-2 RNA improved: the number of PCR amplification instruments increased from 6 to 10 and the number of nucleic acid extractors increased from 3 with 32 channels to 3 with 32 channels and 3 with 96 channels. In addition, the specimens were monitored through a large screen display with a computer screen, and those specimens that had no results since the beginning of sampling for more than 20 h were marked and tested in time. The specimen turn-around process before and after re-engineering in April and July is shown in Figure 1.

### Statistical analysis

All analyses were performed using SPSS software version 19.0 (SPSS, Inc., Chicago, IL, United States). Continuous variables were expressed as mean ± standard deviation (mean ± SD) when the data presented in this study showed a normal distribution. The mean ± SD of two specimens was analyzed by the Independent-Sample *t*-test, and the mean ± SD of more than two samples was compared with the *One-Way ANOVA*. Continuous variables with skewed distribution were presented as median (2.5th to 97.5th percentiles). The median (2.5th to 97.5th percentiles) of two samples was analyzed using the *Mann–Whitney U* and the median (2.5th to 97.5th percentiles) of K-independent samples (more than two samples) was compared with the *Kruskal-Wallis H* test. Categorical variables were presented as a percentage and analyzed using the Chi-Square test. All *p*-values were two-tailed, and a *p*-value <0.05 was considered statistically significant.

## Results

### The main characteristics of subjects 2 weeks before and 2 weeks after the outbreak of COVID-19 in April and July

There was a significant difference in gender proportion and the proportion of women was higher than men (56.38% vs. 43.62%, *p* < 0.001). In different age groups, the proportion of adults aged 19–60 years was the highest (66.38%), followed by children aged 1–6 years (21.43%), and the proportion of newborns less than 1 month old was the lowest (0.42%). There were significant upward trends in the percentage of self-service volunteers through HIS or Tianfu health platform, as well as mixed specimens from April before process re-engineering to July after process re-engineering, which was 0 and 7.18% in the 2 weeks before the COVID-19 outbreak in April to 1.25 and 11.44% in the two 2 after the COVID-19 outbreak in April, and 3.84 and 14.00% in the 2 weeks before the COVID-19 outbreak in July to 24.47 and 36.35% in the 2 weeks after the COVID-19 outbreak in July, respectively (*p* < 0.001). The main characteristics of subjects in the 2 weeks before and the 2 weeks after the two outbreaks of COVID-19 are reported in Table 1.

**Table 1:** The main characteristics of subjects 2 weeks before and 2 weeks after the two outbreaks of COVID-19 in April and July 2022.

	Whole specimen (*n* = 359,845)	Outbreak of COVID-19 in April		Outbreak of COVID-19 in July		*p*-value
		Two weeks before (*n* = 54,416)	Two weeks after (*n* = 78,831)	Two weeks before (*n* = 73,714)	Two weeks after (*n* = 152,884)	
Gender						
Male (%)	43.62	6.59	9.62	9.17	18.24	<0.001
Female (%)	56.38	8.53	12.29	11.32	24.24	<0.001
Age						
< 1 month (%)	0.42	0.09	0.14	0.06	0.13	<0.001
1–12 months (%)	2.88	0.55	0.82	0.47	1.04	<0.001
1–6 years (%)	21.43	3.28	4.93	4.18	9.04	<0.001
7–18 years (%)	6.45	0.95	1.43	1.32	2.75	0.326
19–60 years (%)	66.38	9.91	14.04	13.97	28.46	<0.001
> 60 years (%)	2.44	0.34	0.55	0.49	1.06	0.007
Medical type						
In-patient (%)	5.90	1.20	1.18	1.14	2.38	<0.001
Fever clinic (%)	7.33	1.36	1.35	2.41	2.21	<0.001
General out-patient (%)	44.39	8.71	12.15	11.62	11.91	<0.001
Emergency patient (%)	3.80	0.86	0.75	1.10	1.09	<0.001
Physical examination (%)	9.02	2.99	5.23	0.37	0.43	<0.001
Self-service volunteer through HIS or Tianfu health platform (%)	29.56	0	1.25	3.84	24.47	<0.001
Name of SARS-CoV-2 RNA test item						
Single specimen rapid test (%)	8.85	1.59	1.55	2.89	2.82	<0.001
Single specimen routine test (%)	22.18	6.35	8.92	3.59	3.32	<0.001
Pooling of the specimen test (%)	68.97	7.18	11.44	14.00	36.35	<0.001

### The proportion of specimens of OPS and NPS for detecting SARS-CoV-2 RNA

**Figure 2:**
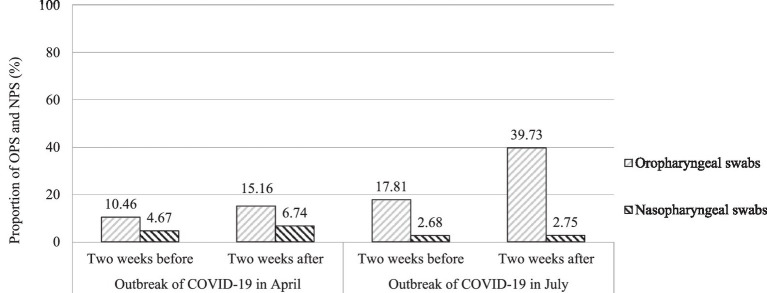
The proportion of OPS and NPS in the 2 weeks before and 2 weeks after the two outbreaks of COVID-19 in April and July.

Among the 359,845 subjects, 83.16% of people chose OPS specimens to detect SARS-CoV-2 RNA, while 16.84% chose NPS specimens. The proportion of OPS had a gradual upward trend from 10.46% in the two 2 weeks before the outbreak of COVID-19 in April to 15.16% within 2 weeks after the outbreak of COVID-19 in April to 17.81% in the 2 weeks before the COVID-19 outbreak in July to 39.73% within 2 weeks after the COVID-19 outbreak in July, respectively (χ^2^ = 31043.44, *p* < 0.001). The proportion of OPS and NPS in the 2 weeks before and 2 weeks after the two outbreaks of COVID-19 in April and July is shown in Figure 2.

### Comparison of the number of specimens in the 2 weeks before and after the two outbreaks of COVID-19 in April and July

**Figure 3:**
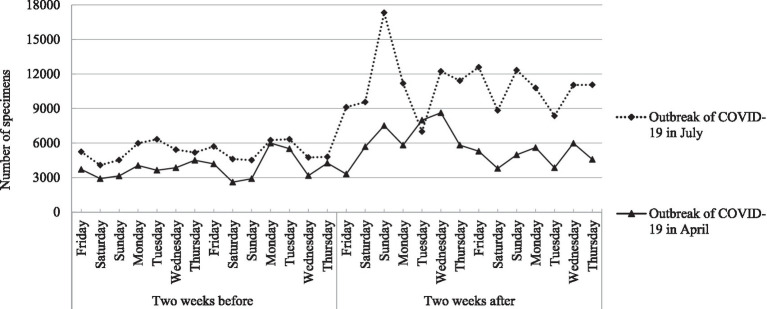
The daily number of specimens in the 2 weeks before and after the two outbreaks of COVID-19 in April and July.

During the non-epidemic period of the 2 weeks before the outbreak of COVID-19 in both April and July, the daily number of specimens on weekends was smaller than on weekdays, which were 2,601–3,139 (0.72–0.87%) on weekends and 3,168–5,594 (0.88–1.67%) on weekdays in April, and 4,077–4,607 (1.13 -1.28%) on weekends and 4,750–17,328 (1.32–1.76%) on weekdays in July, respectively (χ^2^ = 141.63, *p* < 0.001). The detection capability of SARS-CoV-2 RNA has been significantly improved after the process re-engineering, with a maximum of 17,328 specimens detected every day. Compared with April, the number of specimens in July increased significantly not only 2 weeks before (14 days from Friday to Thursday) the outbreak of COVID-19 but also 2 weeks after (14 days from Friday to Thursday) the outbreak of COVID-19, with an increase of 35.46 and 93.94%, respectively (χ^2^ = 2525.73, *p* < 0.001). The daily number of specimens in the 2 weeks before and after the two outbreaks of COVID-19 in April and July is shown in Figure 3.

### Comparison of transportation time and detection time of SARS-CoV-2 RNA specimens before and after process re-engineering in April and July

After the process re-engineering, the number of reports more than 24 h in the 2 weeks before and after the outbreak of COVID-19 in July was significantly lower than that in April before process re-engineering (0% vs. 0.06% (33/54416), χ^2^ = 44.71 and 0 vs. 0.89% (703/78831), χ^2^ = 1367.54, both *p* < 0.001). Whether it is 2 weeks before or 2 weeks after the outbreak of COVID-19, the transportation time of single rapid test specimen, single routine test specimen, and mixed test specimen, as well as the detection time of single routine test specimen and mixed test specimen were significantly lower than those in April (*p* < 0.001). However, there were no significant differences in the detection time of specimens of single rapid test specimen between 2 weeks before the outbreak of COVID-19 in April, 2 weeks after the outbreak of COVID-19 in April, 2 weeks before the outbreak of COVID-19 in July, and 2 weeks after the outbreak of COVID-19 in July (*F* = 1.93, *p* = 0.11). The transportation time and detection time of SARS-CoV-2 RNA specimens before and after process re-engineering in April and July 2022 are shown in Table 2.

**Table 2:** Comparison of transportation time and detection time of SARS-CoV-2 RNA specimens before and after process re-engineering in April and July 2022.

	Transportation time of specimen						Detection time of specimen					
	Single rapid test		Single routine test		Pooling of the specimen test		Single rapid test		Single routine test		Pooling of the specimen test	
	*n*	Mean ± SD	*n*	Mean ± SD	*n*	Mean ± SD	*n*	Mean ± SD	*n*	Median (2.5th–97.5th)	*n*	Median (2.5th–97.5th)
Two weeks before the outbreak of COVID-19 in April	5,726	80.93 ± 35.30	22,845	111.28 ± 38.46	25,845	171.52 ± 65.40	5,726	164.45 ± 36.11	22,845	235.93 (156.56–634.72)	25,845	262.88 (158.37–745.82)
Two weeks after the outbreak of COVID-19 in April	5,562	71.85 ± 33.84^a^	32,103	123.65 ± 42.48^c^	41,166	185.17 ± 68.99^c^	5,562	164.28 ± 37.39	32,103	249.28 (163.62–696.81)^c^	41,166	263.37 (169.54–913.29)
Two weeks before the outbreak of COVID-19 in July	10,389	59.40 ± 29.03^ab^	12,933	82.00 ± 39.47^ab^	50,392	90.72 ± 33.81^ab^	10,389	163.25 ± 34.96	12,933	216.12 (133.47–376.90)^ab^	50,392	222.50 (135.82–375.45)^ab^
Two weeks after the outbreak of COVID-19 in July	10,162	63.48 ± 28.87^abd^	11,934	87.38 ± 42.75^abd^	130,788	91.17 ± 34.57^ab^	10,162	164.09 ± 34.40	11,934	217.05 (131.58–380.32)^ab^	130,788	238.82 (140.31–415.54)^abd^
*p*		<0.001		<0.001		<0.001		0.11		<0.001		<0.001

^a^Significantly decreased compared to 2 weeks before the outbreak of COVID-19 in April, ^b^Significantly decreased compared to 2 weeks after the outbreak of COVID-19 in April, ^c^Significantly increased compared to 2 weeks before the outbreak of COVID-19 in April, ^d^Significantly increased compared to 2 weeks before the outbreak of COVID-19 in July.

## Discussion

As a result of globalization, many of the outbreaks, including the outbreak of COVID-19, have increased the possibility of a pandemic and would pose a burden on society and health systems. For respiratory viral diseases such as COVID-19, early identification and isolation of positive persons is the most effective way to inhibit further human-to-human transmission and mitigation of disease outbreaks (11, 12). As we all know, when there is an outbreak of COVID-19 in an area, rapid large-scale and accurate detection of SARS-CoV-2 RNA involving all people in this area plays a pivotal role in effectively preventing and controlling the further development of COVID-19 (13–15). However, due to the propagation characteristics of SARS-CoV-2 and the bottleneck for testing the presence of the virus (the limitation of detection capability and efficiency), rapid large-scale and accurate detection of SARS-CoV-2 RNA for all people in many areas cannot be completed in a short time (16, 17).

The diagnosis of SARS-CoV-2 infection can be carried out in three different ways, including the determination of the targeted virus RNA genome, virus antigen, and virus antibody (18–20). Nowadays, the detection of virus RNA by RT-PCR is the most widely used detection technique for confirming SARS-CoV-2 infection. As previously reported in many literature, NPS and OPS were the most widely used upper respiratory tract specimens recommended for diagnosing SARS-CoV-2 RNA with RT-PCR (21–23). There was controversy in the literature about the ability of the two sampling methods to detect viruses (24–27). Due to the convenience and lower discomfort, OPS specimens were preferred in large-scale detection (28), which was also consistent with the data from our study. Our research shows that more than four-fifths of people chose OPS specimens to detect SARS-CoV-2 RNA, while less than chose NPS specimens. The proportion of OPS had a gradual upward trend from 10.46% in the 2 weeks before the outbreak of COVID-19 in April to 15.16% within 2 weeks after the outbreak of COVID-19 in April to 17.81% in the 2 weeks before the COVID-19 outbreak in July, to 39.73% within 2 weeks after the COVID-19 outbreak in July, respectively (*p* < 0.001).

Besides the OPS or NPS specimen type, there are still several bottlenecks in our laboratory that limit the capability and efficiency of large-scale detection of SARS-CoV-2 RNA for symptomatic and asymptomatic patients. One of the important bottlenecks is that the number of specimens is too large, which exceeds the test load. Therefore, we have added a test item option of P-10 specimens to use for detecting SARS-COV-2 RNA, which is selected by volunteers under the doctor’s order. In addition, P-10 specimens can be quickly sampled by scanning for personal information on the Tianfu Health Code in Sichuan. Our results showed that through process re-engineering, there were significant upward trends in the percentage of P-10 specimens from April before process re-engineering to July after process re-engineering, which was 7.18% in the 2 weeks before the COVID-19 outbreak in April, to 11.44% in the 2 weeks after the COVID-19 outbreak in April, and 14.00% in the 2 weeks before the COVID-19 outbreak in July, to 36.35% in the 2 weeks after the COVID-19 outbreak in July, respectively (*p* < 0.001). Compared with April, the number of specimens in July increased significantly not only 2 weeks before the outbreak of COVID-19 but also 2 weeks after the outbreak of COVID-19, with an increase of 35.46 and 93.94%, respectively (*p* < 0.001). The maximum number of specimens detected per day was 17,328. If there is no P-10 specimens option, more hardware and human resources would be invested every day to complete so many tests.

The P-10 strategy saves time and resources but it also has limitations. As various studies showed, test sensitivity was inversely proportional to testing efficiency depending on pool size (29). In 2021, the Department of Medical Affairs and Medical Management of the National Health Commission issued the “Guidelines for the Implementation of All Citizens Novel Coronavirus Nucleic Acid Organization,” which explicitly suggested that the P-10 strategy should be used for large-scale screening of the population. The guidelines clearly stated: (1) pooling of 10 samples strategy: the first round of testing can all be done using the P-10 strategy to screen out infected persons as quickly as possible. Subsequently, the whole population can be screened by nucleic acid testing in accordance with the program of single-collection testing in key populations and high-risk areas.

Another bottleneck in our laboratory is the lower matching of information exchange between HIS and LIS due to the fact that the LIS and HIS software are produced by different software companies. For this purpose, the whole process of informatization was re-engineered through multi-department cooperation. Firstly, we changed the HIS sampling procedure to the LIS sampling procedure, which better matches the follow-up LIS specimens transportation, reception, test, report dispatch, etc., and greatly facilitates the traceability of specimens and the search of problem specimens. Secondly, we carried out packaging management for multi-tube specimens by a packaging barcode and quickly obtained the information of everyone in all packaged specimen tubes by scanning the packaging barcode, which greatly improved the work efficiency. Thirdly, we added a scanning code (LIS barcode or Tianfu Health Code) program at the sampling site, and the sampling time of each specimen was obtained by scanning the code during sampling. At the same time, most problem barcodes that could not be scanned or the problem specimens without barcodes could not enter the laboratory, which helped PCR testers avoid spending a lot of time and effort to deal with these problem barcodes or problem specimens.

In addition, some hardware, such as the number of PCR amplification instruments and the number and channel of nucleic acid extractors, limited the detection capability and efficiency of our laboratory. Before process re-engineering, there were six PCR amplification instruments and three nucleic acid extractors with 32 channels in our laboratory. After process re-engineering, we added four PCR amplification instruments and three nucleic acid extractors with 96 channels. This way, even if some instruments fail, the detection capability and efficiency of SARS-CoV-2 RNA will not be affected. Furthermore, we changed the frequency of specimen transportation. Emergency specimens were transported once within 30 min and routine specimens were transported once within 2 h. When the number of specimens was large, they were transported once within 1 h. At the same time, we added a large screen display to monitor the specimens sampled for more than 20 h without results, which were tested timely because the Tianfu health platform of Sichuan requires that TAT should not exceed 24 h for all SARS-CoV-2 RNA specimens.

In the present study, in the non-epidemic period of the 2 weeks before the outbreak of COVID-19, whether in April or July, the daily number of specimens on weekends was smaller than on weekdays (*p* < 0.001). In order to reduce the difference in data between weekdays and weekends, we selected the date of the first outbreak of COVID-19 in April and July (both on Friday) as the time node and selected the data 2 weeks before the outbreak of COVID-19 (Friday to Thursday) and 2 weeks after the outbreak of COVID-19 (Friday to Thursday) as the data of this study. Our data showed that through process re-engineering, whether it was 2 weeks before or 2 weeks after the outbreak of COVID-19, the transportation time of the single rapid test specimen, single routine test specimen, and mixed test specimen, as well as the detection time of single routine test specimen and mixed test specimen, were significantly lower than those in April (*p* < 0.001). Moreover, after the process re-engineering, the number of reports of more than 24 h in the 2 weeks before and after the outbreak of COVID-19 in July was significantly lower than that in April before process re-engineering (0% vs. 0.06% and 0 vs. 0.89%, both *p* < 0.001).

### Limitations

Since this process re-engineering was only a single site study, and the investigation period was short with only 2 weeks before and 2 weeks after the two outbreaks of COVID-19 and the number of specimens was smaller, a larger number of specimens in a multicenter study and longer investigation period are necessary to further confirm the carrying capacity of network and server when a lot of people access Tianfu health platform in a centralized manner in a large-scale detection of SARS-COV-2 RNA.

## Conclusion

This study demonstrated that more than four-fifths of people chose OPS specimens to detect SARS-CoV-2 RNA, while less than one-fifths chose NPS specimens. Strengthening the cooperation of multi-departments in the process re-engineering, especially using P-10 specimens and whole process informatization, can improve the detection capability and efficiency of SARS-CoV-2 RNA specimens. Adding a large screen monitor to monitor specimens for more than 20 h without results can effectively prevent specimens from TAT for more than 24 h. We believe that process re-engineering can be used to detect not only SARS-CoV-2 RNA but probably also other pathogenic microorganisms during the outbreak.

## Data availability statement

The raw data supporting the conclusions of this article will be made available by the authors, without undue reservation.

## Ethics statement

The studies involving humans were approved by this study was approved by the Medical Ethics Committee at Chengdu Women’s and Children’s Central Hospital, Chengdu, China [(2019)126, Medical Ethics Committee, CWCCH]. Written informed consent for participation or for the publication of any potentially identifiable images or data included in this article was not required from the participants or the participants’ legal guardians/next of kin because the present study is a retrospective cross-sectional study. However, the private information in the study has been well protected.

## Author contributions

CL: Writing – original draft, Writing – review & editing. WS: Data curation, Investigation, Writing – review & editing. HX: Software, Writing – review & editing. YinL: Methodology, Writing – review & editing. RC: Project administration, Writing – review & editing. RW: Visualization, Writing – review & editing. LX: Data curation, Supervision, Writing – review & editing. JL: Project administration, Validation, Writing – review & editing. YG: Investigation, Project administration, Writing – review & editing. YiL: Project administration, Software, Writing – review & editing. CZ: Project administration, Validation, Writing – review & editing. YX: Data curation, Formal analysis, Writing – review & editing. QW: Conceptualization, Writing – review & editing, Resources.
